# Realizing the promise of ‘La Dolce Vita’ via chemical biology: glycan‐motif editing of sLe^X^ for precision cancer therapeutics

**DOI:** 10.1111/febs.70079

**Published:** 2025-03-27

**Authors:** Barbara Richichi, Robert Sackstein

**Affiliations:** ^1^ Department of Chemistry ‘Ugo Schiff’ University of Firenze Italy; ^2^ Department of Translational Medicine and Translational Glycobiology Institute, Herbert Wertheim College of Medicine, Florida International University Miami FL USA

**Keywords:** E‐selectin, E‐selectin ligand, fucosyltransferase, fucosyltransferase inhibitor, glycan‐motif editing, glycocalyx, glycocalyx editing, glycomimetics, sLe^X^, translational glycobiology

## Abstract

The convergence of glycochemistry and glycobiology is enabling the creation of new therapeutic approaches with unprecedented capacity to alter cell and organismic biology using strategies that can uniquely and specifically custom‐modify the expression of key cell surface glycan motifs. We define this evolving field of chemical biology as ‘glycan‐motif editing’, and one of the principal targets of this glycoengineering effort is the sialofucosylated terminal lactosaminyl glycan known as sLe^X^ (CD15s). This tetrasaccharide structure plays pivotal roles in both steady‐state and malignant hematopoiesis, in regulation of the immune response, and in cancer metastasis. Within this biological framework, we discuss the immense potential of glycan‐motif editing in enabling precision therapeutics that will profoundly improve outcomes for patients suffering from a wide variety of disabling and life‐threatening conditions, particularly cancer.

## Graphical abstract

Modification of the glycocalyx via ‘glycan editing’ is distinguishable from ‘glycan‐motif editing’. The former broadly remodels the glycocalyx, whereas the latter inhibits expression only of a specific oligosaccharide motif within the glycocalyx by selectively dampening the bioactivity of the key glycosyltransferase(s) programming the biosynthesis of the pertinent oligosaccharide. As shown, prevention of cellular expression of sialyl Lewis X (CD15s), a driver of carcinogenesis and metastasis, is achievable via a small‐molecule inhibitor uniquely targeting α(1 → 3)‐fucosyltransferases.

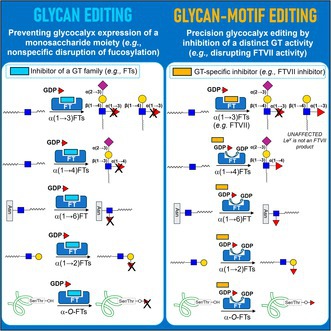

**Abbreviations**
FTs: fucosyltransferases
GTs: glycosyltransferases
Le^X^
: Lewis X
sLe^a^
: sialylated Lewis A
sLe^X^
: sialylated Lewis X
STs: sialyltransferases
TACA: tumor associated carbohydrate antigens

## Introduction

Glycosylation is a major post‐translational modification [1] that results in the attachment of glycans (complex carbohydrates) to other biomolecules, such as protein and lipids to create ‘glycoconjugates’ (*i.e*., glycoproteins and glycolipids, respectively). Essentially, all cell membrane proteins and lipids are glycoconjugates, and, as such, the composition of the surface of any given cell reflects a sum total of the glycan structures displayed on pertinent protein and/or lipid scaffolds. This meshwork of glycans is called the ‘glycocalyx’. The glycocalyx influences how cells interact with their environment, thereby regulating various biological processes critical to cell/tissue homeostasis and immune responses, as well as to the pathobiology of inflammation, infectious diseases, and cancer [1]. These processes are dictated by the expression of distinct oligosaccharide structures within the glycocalyx called ‘glycan motifs’. These motifs are comprised of discrete monosaccharides assembled via regio‐ and stereospecific covalent bond linkages that are orchestrated by the action(s) of specific glycosyltransferases (GTs) [2, 3, 4, 5].

**Fig. 1:**
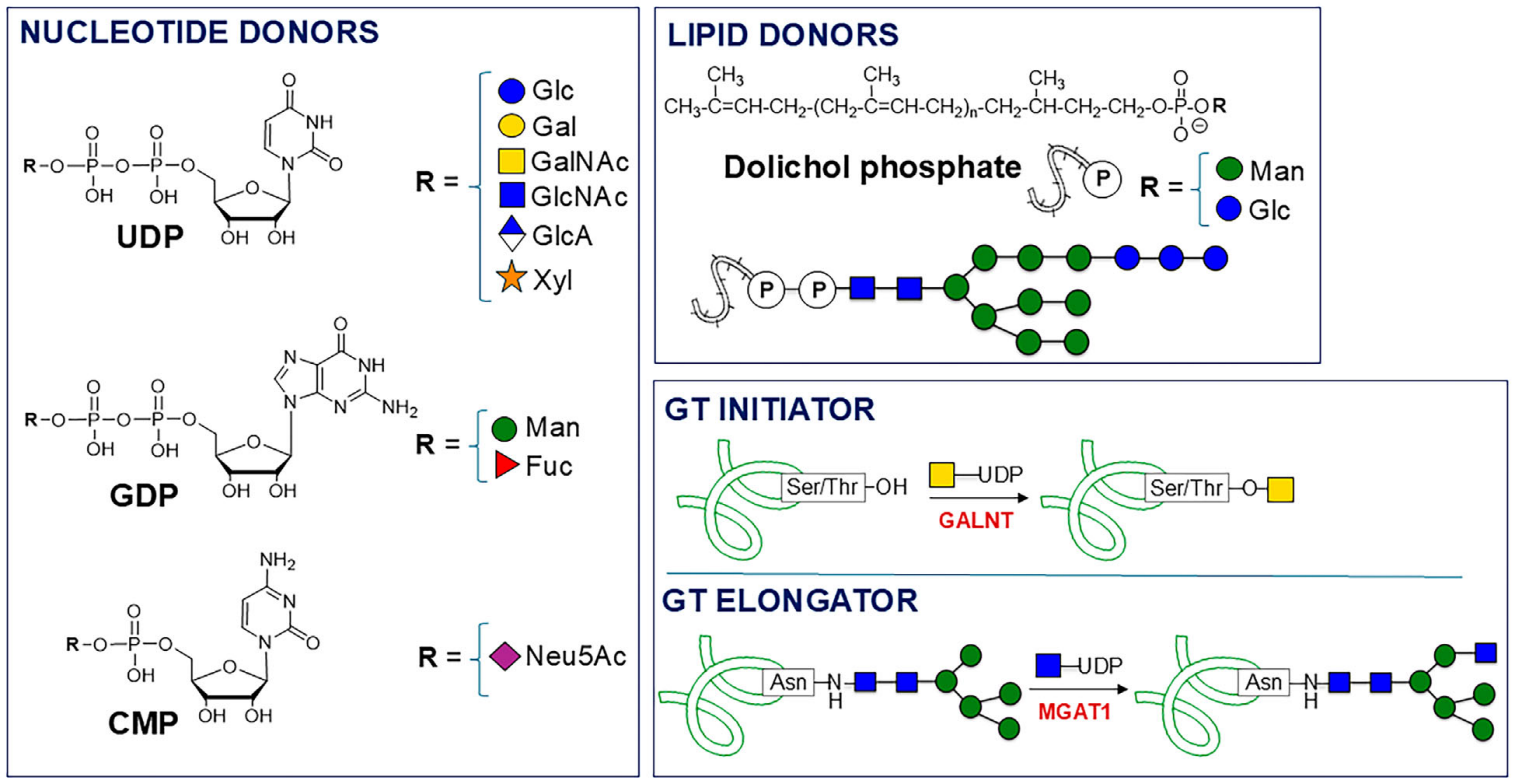
Structures of the activated donors used by most of the human glycosyltransferases (GTs). Nucleotide‐linked monosaccharides (nucleotide donors): d‐glucose (Glc), d‐galactose (Gal), d‐*N*‐acetyl‐galactosamine (GalNAc), d‐*N*‐acetyl‐glucosamine (GlcNAc), d‐glucuronic acid (GlcA), and d‐xylose (Xyl) are activated as uridine diphosphate (UDP) donors; d‐mannose (Man) and l‐fucose (Fuc) are activated as guanosine diphosphate (GDP) donors; *N*‐acetyl‐neuraminic acid (Neu5Ac) is activated as a cytidine monophosphate (CMP) donor. Lipid‐linked mono‐ and oligosaccharides (lipid donors): Man and Glc are activated as donors linked to dolichol phosphate; other GTs use as donor substrate an oligosaccharide chain linked to a dolichol‐diphosphate (*e.g*., as it occurs in the biosynthesis of *N*‐glycans in the endoplasmic reticulum (ER)). Representative examples of a ‘GT initiator’ (α‐*N*‐acetyl‐galactosaminyltransferase, ‘GALNT’) that catalyzes the formation of an *O*‐glycosidic linkage with the serine or threonine residue of a protein (biosynthesis of *O*‐glycans), and of a ‘GT elongator’ (β‐*N*‐acetyl‐glucosaminyltransferase, ‘MGAT1’) that catalyzes the formation of an *N*‐glycosidic linkage with the asparagine residue of a protein (biosynthesis of *N*‐glycans) [6].

The endoplasmic reticulum (ER) and the Golgi apparatus are the primary sites of protein and lipid glycosylation in eukaryotic cells. GTs are mainly located in these organelles where they catalyze the sequential glycosidic attachment of monosaccharides (donor substrates) to specific acceptor molecules (*i.e*., carbohydrates, proteins, and lipids). Accordingly, GTs are classified into two categories: ‘initiator GTs’ that attach the first monosaccharide of an oligosaccharide chain to a protein (*e.g*., via linkage to nitrogen in asparagine (*i.e*., ‘*N*‐glycans’) or to oxygen in serine or threonine (*i.e*., ‘*O*‐glycans’)) or to a lipid acceptor, and ‘elongator GTs’ that extend glycan chains by adding additional monosaccharides (Fig. 1). GTs catalyze this transfer using ‘activated’ donor substrates, *i.e.* nucleotide‐linked monosaccharides (most commonly monosaccharides covalently linked to either uridine diphosphate (UDP), guanosine diphosphate (GDP) or cytidine monophosphate (CMP)), or as lipid‐linked oligosaccharides or monosaccharides covalently linked to dolichol phosphate (Fig. 1) [6].

Assembled glycans can, and most frequently do, undergo further modifications through the selective removal of monosaccharides by enzymes named glycosidases. As a result, any glycan motif displayed on a pertinent cell surface represents a balance between GT‐mediated biosynthesis and subsequent glycosidase‐mediated degradation. But, fundamentally, since the glycan motif has been constructed by virtue of GT‐mediated assembly of the component monosaccharides, the ability to regulate expression and/or activity of GTs is key to performing precision glycocalyx editing: in particular, to custom‐modify the expression of specific motifs to achieve intended therapeutic aims, a technology we call ‘glycan‐motif editing’.

## Cancer‐associated changes in the Glycocalyx

Post‐translational glycosylations require considerable consumption of a cell's metabolic energy, and thus, glycan modifications would not otherwise occur unless they were to confer a net advantage to that cell's biologic activity and/or viability. Accordingly, changes in cell surface glycan composition are not random, and the expression of glycan determinants is a fingerprint of specific diseases, including cancer [7, 8, 9, 10]. Therefore, the identification of the structure and composition of such glycans, coupled with knowledge regarding the control of their biosynthesis, paves the way for the discovery of disease biomarkers as well as the development of precision therapeutics. The understanding of disease‐associated glycosylations is crucial for creating target‐specific therapeutic interventions that rely on the manipulation of glycosylation, thus offering promising avenues to cure a wide variety of disabling and life‐threatening conditions.

Alterations in the structure and occurrence of glycans is a hallmark of cancer [8]. Changes in the cancer cell glycocalyx (*e.g*., increased sialylation [11, 12], truncated *O*‐glycans [13, 14], altered *N*‐glycan branching [15], and fucosylation changes [16]) are dictated by cancer‐associated modulation of cellular glycan biosynthetic networks. The resultant expression of discrete glycocalyx motifs then further serve to fuel oncogenic transformation and cancer progression [10]: expression of these ‘aberrant’ glycan motifs (‘tumor associated carbohydrate antigens’ (TACA)) within the glycocalyx helps tumors to grow, spread, resist therapy, and evade the immune response [7, 8, 9]. Therefore, cancer‐associated cell surface glycan moieties represent not only biomarkers, but they are also key drivers of cancer development and of the metastatic propensity of the pertinent cancer. By precision editing of these glycan motifs, it may be possible to disrupt these cancer‐promoting mechanisms and provide highly effective therapeutic interventions without significant adverse consequences.

With very few exceptions (*e.g*., CNS tumors), a patient with cancer does not succumb to the effect of the primary tumor itself; the patient succumbs to the pathobiologic consequences of metastasis of the primary tumor. Indeed, metastasis is a major problem in most solid and hematological malignancies. Importantly, leukemia is the most metastatic malignancy: this disease begins as focal proliferation of malignant cells within a defined microenvironment of the marrow at a distinct anatomic site (which can be anywhere within the skeleton) and then disseminates rapidly to the marrow throughout the patient's body. Thus, the patient's entire marrow soon gets occupied by leukemia cells, completely overtaking the healthy hematopoietic cells, and the patient is then unable to make normal blood elements. Viewed in this light, it is critically important to characterize a malignant cell's surface glycan motifs that confer the biologic advantage to metastasize and proliferate at distant sites and, thereafter, to develop pertinent strategies to blunt their expression.

## Distinguishing ‘glycan editing’ versus ‘glycan‐motif editing’

**Fig. 2:**
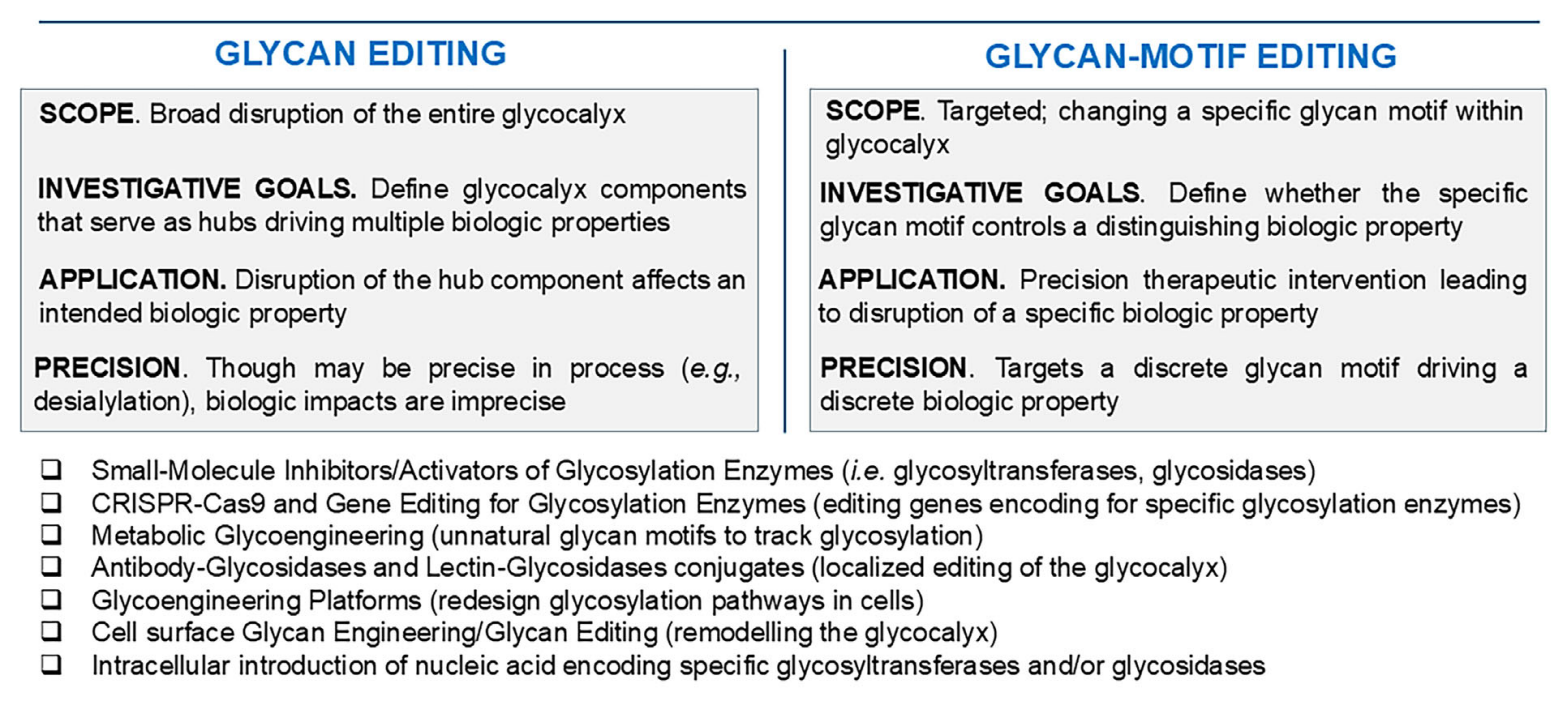
Modulation of expression of glycocalyx components: glycan editing vs glycan‐motif editing.

This article will not cover strategies to remodel the glycocalyx by introducing specific glycan motifs via chemical or chemoenzymatic attachment of distinct monosaccharides and/or oligosaccharides to the cell surface, as these approaches have been recently reviewed [17, 18], and their effects on biological events have been reported [19, 20, 21]. Instead, we will focus here on strategies to remodel the glycocalyx via disrupting the expression of glycan motifs, whereby there are two main strategies (Fig. 2): the broad (wide‐ranging) ‘glycan editing’ approach that disrupts multiple types of glycan motifs embedded within the glycocalyx, and the discriminatory approach of ‘glycan‐motif editing’ which results in the selective interruption in construction of a discrete cell surface oligosaccharide structure. Glycan editing and glycan‐motif editing are closely related concepts in the field of translational glycobiology, indeed, both involve the manipulation of glycocalyx structures to deliberately shape cell biology. However, they differ in the scope, precision, and specificity of the editing. Glycan editing includes strategies that involve changes to the entire glycocalyx, including altering the length of glycan chains, modifying branching patterns, or broadly adding or removing specific monosaccharides (*e.g*., removal of cell surface sialic acid moieties by treating cells with sialidases). As such, glycan editing modifies the overall glycosylation profile of the cell. In contrast, glycan‐motif editing refers to more precise and targeted tactics, focused on modifying specific oligosaccharide domains within a pertinent (larger) polysaccharide structure. The purpose of glycan‐motif editing is to modify the display of specific oligosaccharide moieties that play crucial roles in biological interactions, such as in programming of cell–cell communication, of immune responses, of pathogen binding, and, in the case of cancer, of malignant cell proliferation and metastasis. Furthermore, by focusing on a distinct, regio‐ and stereospecific modulation of a restricted glycocalyx domain, glycan‐motif editing can lead to the creation of novel high precision, personalized therapeutic interventions. However, despite the fact that glycan‐motif editing holds promise in manipulating glycan structures with selectivity, thereby minimizing side effects (*i.e*., ‘off target effects’) and improving therapeutic efficacy, it is technically more complex than glycan editing and requires a detailed knowledge of the biosynthesis of the glycan motif as well as the structural biology of the target oligosaccharide moiety and its role within the pathobiologic process of interest.

Understanding the differences between glycan editing and glycan‐motif editing enables researchers and clinicians to choose the right tool(s)/agent(s) for the specific biologic/therapeutic need(s) (Fig. 2). Importantly, in modifying glycocalyx components, it is critical to appreciate the spectrum of context‐specific glycosylation (*e.g*., glycosylation may vary by cell type, tissue, disease state, and species) and the potential of off‐target effects within the complexity of glycosylation pathways (*e.g*., modifying glycosylation in one pathway could unintentionally affect other critical biological processes, thereby leading to side effects).

## The case study: The biosynthesis of sLe^X^ and the impact of sLe^X^ /E‐selectin interactions on health and carcinogenesis

**Fig. 3:**
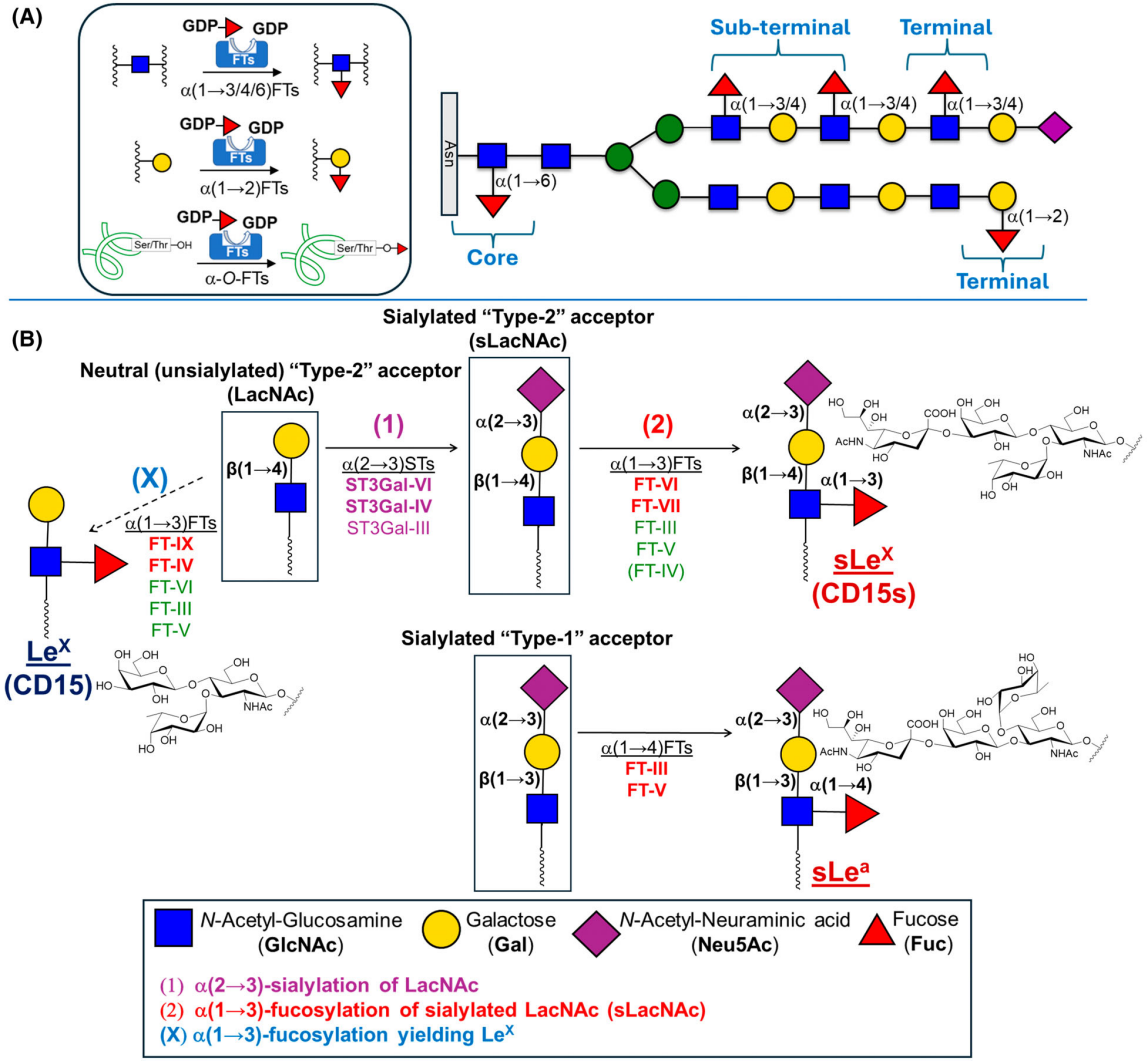
Fucosyltransferase‐mediated reactions. (A) (*Left*) Schematic representation of the fucosylation reactions catalyzed by fucosyltransferases (FTs), and (*Right*) Schematic representation of the various α(1 → 6)‐, α(1 → 3/4)‐, and α(1 → 2)‐ fucosylation sites (*i.e*., *core*, sub‐terminal and terminal) showing, as an example, the glycan acceptor(s) of a protein *N*‐glycan (with exception of *core* fucose modification on *N*‐glycans, similar reactions target respective glycan acceptors displayed on protein *O*‐glycans as well as on glycans of glycolipids); (B) Golgi glycosyltransferases that mediate biosynthesis of Le^X^ (CD15) and of sLe^X^ (CD15s) showing pertinent α(1 → 3)‐FTs that fucosylate the LacNAc (Gal‐β(1 → 4)‐GlcNAc‐β‐1‐R (named as neutral (unsialylated)‐‘Type‐2’ acceptor) or sLacNAc (Neu5Ac‐α(2 → 3)‐Gal‐β(1 → 4)‐GlcNAc‐β‐1‐R (named as sialylated ‘Type‐2’ acceptor) structures respectively, and of sLe^a^ showing pertinent α(1 → 4)‐FTs (*i.e*., FTIII and FTV) that fucosylate the Neu5Ac‐α(2 → 3)‐Gal‐β(1 → 3)‐GlcNAc‐β‐1‐R (named as sialylated ‘Type‐1’ acceptor). For each reaction, the potency of the respective glycosyltransferases (GTs) is listed in order (GT with highest potency is placed at the top) and red bold fond indicates the α(1 → 3/4)‐FTs most potent in mediating creation of Le^X^, sLe^X^ or sLe^a^. ‘(X)’ denotes that α(1 → 3)‐fucosylation of LaNAc creates Le^X^, preventing creation of sLe^X^ from a LacNAc precursor.

Fucosyltransferases [2, 22, 23, 24, 25, 26] are a family of 13 enzymes that transfer an l‐fucose (Fuc) residue from a donor substrate guanosine diphosphate‐fucose (GDP‐fucose, GDP‐Fuc) to a pertinent acceptor via α(1 → 2)‐linkage to a d‐galactose (Gal), or via α(1 → 3/4)‐ or α(1 → 6)‐linkages to a d‐*N‐*acetyl‐glucosamine (GlcNAc) on a large array of *N*‐ and *O*‐glycans, or via *O*‐fucosylation to serine/threonine residues within epidermal growth factor (EGF)‐like, thrombospondin type 1 repeat domains and, as recently discovered [26], within the elastin microfibril interface (EMI) domain of Multimerin‐1 (MMRNI) (Fig. 3A). According to the position of the Fuc on the glycan chain (*i.e*., fucosylation site, Fig. 3A), we can distinguish between terminal (α(1 → 2) and α(1 → 3/4)‐linkages), sub‐terminal (α(1 → 3/4)‐linkages), and *core* (α(1 → 6)‐linkage) fucosylations (Fig. 3A).

Among the FT family, there are six human α(1 → 3)‐FTs (*i.e*., FTIII, FTIV, FTV, FTVI, FTVII, and FTIX) that catalyze the transfer of a Fuc residue to the GlcNAc residue on saccharide units located at the termini of glycan chains (Fig. 3B), *i.e.* on either Gal‐β(1 → 4)‐GlcNAc‐β‐1‐R (named as neutral (unsialylated)‐‘Type‐2’ acceptor, LacNAc,) or Neu5Ac‐α(2 → 3)‐Gal‐β(1 → 4)‐GlcNAc‐β‐1‐R (named as sialylated ‘Type‐2’ acceptor, sLacNAc). Each α(1 → 3)‐FT enzyme exhibits specificity for distinct acceptor substrates, and they shape the biosynthesis of different glycan determinants collectively known as ‘Lewis antigens’ [27].

The α(1 → 3)‐fucosylated sialyllactosaminyl glycan known as ‘sialylated Lewis X’ (sLe^X^ (CD15s): Neu5Ac‐α(2 → 3)‐Gal‐β(1 → 4)‐[Fuc‐α(1 → 3)]‐GlcNAc‐β‐1‐R, Fig. 3B) serves as a prime example of how the installation of a single monosaccharide (*i.e*., the l‐Fucose), in stereo‐ and regiospecific linkage to its pertinent acceptor glycan controls a plethora of biologic events. The sLe^X^ motif is displayed as a ‘terminal’ structure, localized at the nonreducing end of glycans displayed on either glycoproteins (either as *O*‐glycans or *N*‐glycans) or on glycolipids. Its assembly requires the coordinated and sequential action of two types of Golgi glycosyltransferases, an α(2 → 3)‐sialyltransferase (α(2 → 3)‐ST) and an α(1 → 3)‐fucosyltransferase (α(1 → 3)‐FT) working sequentially, in each case modifying a terminal ‘Type 2’ lactosamine backbone: Gal‐β(1 → 4)‐GlcNAc‐β‐1‐R (LacNAc). As shown in Fig. 3B, assembly of the sLe^X^ tetrasaccharide from the disaccharide LacNAc core proceeds first by the terminal addition of a sialic acid (also known as ‘*N*‐acetyl‐neuraminic acid’; abbreviated as ‘Neu5Ac’) in α(2 → 3)‐linkage to a Gal residue to create the trisaccharide known as a sialylated ‘Type‐2’ lactosamine (Neu5Ac‐α(2 → 3)‐Gal‐β(1 → 4)‐GlcNAc‐β‐1‐R (sLacNAc)). Three members of the α(2 → 3)‐ST family can catalyze this reaction (ST3Gal‐III, ST3Gal‐IV, and ST3Gal‐VI), following which addition of a Fuc residue (from GDP‐fucose) via α(1 → 3)‐linkage to the GlcNAc residue is catalyzed by specific α(1 → 3)‐FT isoenzymes (mainly FTVI and FTVII and to lesser extent by FTIII and FTV and, very minimally, by FTIV, Fig. 3B) thus yielding sLe^X^ [2, 25, 27]. The creation of sLe^X^ must follow this fixed, sequential order of reactions, as there are no α(2 → 3)‐STs that can link a sialic acid (Neu5Ac) to Gal within an α(1 → 3)‐fucosylated ‘Type‐2’ lactosamine; this (unsialylated) trisaccharide structure is called ‘Lewis X’ (Le^X^; CD15), wherein the α(1 → 3)‐fucosylation is principally mediated by FTIX and FTIV (Fig. 3B).

Upon installation of fucose to create the α(1 → 3)‐fucosylated tetrasaccharide sLe^X^ onto a corresponding glycoconjugate, leukocytes gain license to a range of functions, including immunosurveillance and immunoreactivity, enabling not only cell homing, but signal transduction, cell growth, and adhesion [28]. sLe^X^, along with its α(1 → 4)‐fucosylated ‘Type 1’ sialylated isomer known as ‘sialylated Lewis A’ (sLe^a^: Neu5Ac‐α(2 → 3)‐Gal‐β(1 → 3)‐[Fuc‐α(1 → 4)]‐GlcNAc‐β‐1‐R) (Fig. 3B), comprises the canonical binding determinants for E‐selectin [29, 30]. The α(1 → 4)‐fucosylation of GlcNAc within a terminal sialylated ‘Type 1’ acceptor is catalyzed by either one of two FTs, FTIII and FTV; as such, FTIII and FTV are distinguished by serving as both α(1 → 3) FTs and α(1 → 4)‐FTs (hence, they are ‘α(1 → 3/4)‐FTs’). Notably, sLe^a^ is best‐recognized by the name CA19‐9, a clinically useful biomarker of mucinous cancers (*e.g*., gastrointestinal malignancies, including pancreatic cancer and cholangiocarcinoma) and a principal mediator of hematogenous metastasis of such cancers [31].

E‐selectin (CD62E) is a member of the selectin family of Ca^2+^‐dependent lectins, which contains two other members: L‐selectin (CD62L) and P‐selectin (CD62P) [32, 33]. Lectins are proteins that bind to sugars, and, as the name implies, E‐selectin is an endothelial lectin (hence, ‘E‐’). E‐selectin is expressed constitutively on microvessels of the skin and marrow, and, notably, its endothelial expression is otherwise transcriptionally upregulated by inflammatory cytokines (principally TNF and IL‐1), by all forms of trauma, by ischemia, and by microbial products (*e.g*., LPS) at post‐capillary venules within affected endothelial beds at all sites of tissue injury/inflammation. E‐selectin is also characteristically expressed within endothelial beds of tumors, in large part by induction secondary to TNF.

E‐selectin binds to sLe^X^ and to sLe^a^. sLe^X^‐decorated glycoconjugates are expressed on human leukocytes, on human hematopoietic progenitor cells, and on cells of both hematologic and nonhematologic malignancies. E‐selectin receptor–ligand interactions are the most potent effectors of Step 1 of the multistep cascade of cell migration and also mediate lodgment of cells within tissue microenvironments [34, 35, 36]. Thus, E‐selectin‐sLe^X^ receptor/ligand interactions are critical for regulating trafficking of leukocytes to sites of tissue injury/inflammation, for regulating both physiologic and pathobiologic blood cell formation (hematopoiesis), and for mediating metastasis and growth of malignancies, both hematologic and nonhematologic [19, 37, 38, 39, 40, 41].

Notably, unlike sLe^X^, sLe^a^ expression is restricted to mucinous cells and tumors derived therefrom [8]. However, like sLe^X^, sLe^a^ is a mediator of mucinous cancer metastasis and growth. sLe^a^ is not natively expressed on human leukocytes or on any human hematopoietic progenitor (healthy or malignant) because these cells altogether lack expression of pertinent β(1 → 3)‐galactosyltransferase (*e.g*., β3GalT‐5) that creates terminal Type 1 chain. Thus, though interruption of sLe^a^ biosynthesis via modulating α(1 → 4)‐fucosylation may be leveraged to improve outcomes in solid tumors, there is no role for targeting FTIII‐ or FTV‐mediated α(1 → 4)‐fucosylation with regard to therapeutic shaping of host defense processes, hematopoietic events (including hematologic malignancies), and/or immunopathologic reactions.

The role of E‐selectin/sLe^X^ adhesive interactions in the pathobiology of cancer is well‐recognized, especially regarding solid tumor metastasis [41, 42] and leukemogenesis [43]. sLe^X^ is characteristically expressed on leukemic blasts, and sLe^X^/E‐selectin adhesive interactions drive dissemination (metastasis) of these cells. Furthermore, sLe^X^/E‐selectin adhesive interactions also fuel malignant hematopoiesis via the formation of critical microenvironmental leukemic niches, and this topic has been recently reviewed [42, 43].

There is increasing knowledge regarding the role of sLe^X^ in the onset and invasiveness of solid cancers. Indeed, sLe^X^ is a principal mediator of metastasis of epithelial cancers (which include mucinous cancers), and importantly, the very first serum biomarker of epithelial cancers was FTVI itself [44, 45, 46, 47]. FTVI has been identified as the key FT involved in sLe^X^ biosynthesis in colon cancer tissues, but it is not characteristically expressed in normal mucosa (which has low to no expression of sLe^X^) [48]. Tumor cell sLe^X^ display is also associated with the metastatic propensity of gastrointestinal cancers, and with increased invasiveness of gastric cancer cells [49, 50], through the activation of specific invasiveness‐associated signaling pathways [51], and the aberrant glycosylation [52] of the receptor tyrosine kinase RON (Recepteur d'Origine Nantais). Moreover, clinical studies in breast cancer patients have revealed differences in the expression of sLe^X^ within primary tumor cells compared with cells at metastatic sites: sLe^X^ expression is weak in carcinomas *in situ* whereas metastasized breast cancer cells have uniformly high sLe^X^ levels [53] and serum sLe^X^ levels correlate with the extent of metastasis in breast cancer patients [54]. Furthermore, a study including a cohort of 26 primary tumors from breast cancer patients with skin metastases confirmed the role of sLe^X^ as the driver of breast cancer cutaneous metastases [55]. The relationship between increased sLe^X^ expression and metastatic activity may be fueled by increased endothelial E‐selectin expression within premetastatic niches, as has been described in lung cancer metastasis models [56]. Notably, E‐selectin is constitutively expressed in marrow microvessels, thereby serving as a beacon for the recruitment of sLe^X^‐bearing metastatic cancer cells [40]. For this reason, bone metastasis is extremely common for many malignancies (*e.g*., prostate, breast, and lung), and, in particular, it is well established that the expression of α(2 → 3)‐sialyltransferases and of α(1 → 3)‐FTs (especially FTVI) are each associated with increased metastatic activity of prostate cancer cells [57, 58, 59, 60, 61]. Recently, elevated levels of FTVII and sLe^X^ were detected in muscle‐invasive bladder cancer (BLCA) and were correlated to the growth and invasion of BLCA cells promoted by the increased blood vessel permeability of sLe^X^‐enriched small extracellular vesicles derived from BCLA [62].

Given these facts, interfering with malignant cell sLe^X^‐endothelial E‐selectin interactions by targeted inhibition of sLe^X^ expression and/or by use of sLe^X^‐mimetics that act as competitive inhibitors has drawn increasing attention. With regard to the former approach, the identification of small‐molecule inhibitors of FT enzymes has attracted significant interest. Several approaches have been undertaken thus far, and they have been recently reviewed [23, 63, 64, 65, 66, 67, 68, 69]. Most of the compounds identified to date provide a broad, FT‐family directed glycan editing of all fucosylated glycan determinants, including sLe^X^. However, the manipulation of the entire fucosylation machinery can have significant biological implications. Therefore, the specific catalytic activity of the FTs, and, in particular, the role of α(1 → 3)‐FT isoenzymes in the creation of sLe^X^, should be carefully taken into account in the evaluation of the biological impact of the glycan editing vs the glycan‐motif editing effect mediated by the proposed synthetic inhibitors.

## Applying ‘glycan‐motif editing’ to fine‐tune sLe^X^ expression: Achievables and future perspectives

In studying the cell surface glycan editing properties of small‐molecule inhibitors of GTs, it is important to recognize that lectin probe‐based studies and/or mass spectrometry, albeit they are conventional tools/techniques in glycobiology, are neither as sensitive nor as specific as flow cytometry‐based analysis using glycan motif‐specific monoclonal antibodies (mAb) that selectively target the pertinent oligosaccharide determinant (*i.e*., an ‘anti‐glycan motif mAb’). With regard to sLe^X^, the mAb CLEX‐1 has exquisite specificity for sLe^X^, while the HECA452 mAb recognizes both sLe^X^ and sLe^a^. However, both antibodies are useful for discriminating the effectiveness of inhibition of α(1 → 3)‐FTs (*e.g*., inhibition of FTVI) because, in each case, inhibition of α(1 → 3)‐fucosylation will blunt expression of sLe^X^. The ability to utilize flow cytometry to accurately quantify the level of expression of sLe^X^ motifs present within the cell's glycocalyx (*i.e*., on the cell's surface) before and after treatment with a pertinent inhibitor of an α(1 → 3)‐FTs is a major technical advantage, a key contributor to achieving the superior sensitivity and specificity in the use of any given anti‐glycan motif mAb in measuring its target determinant.

**Fig. 4:**
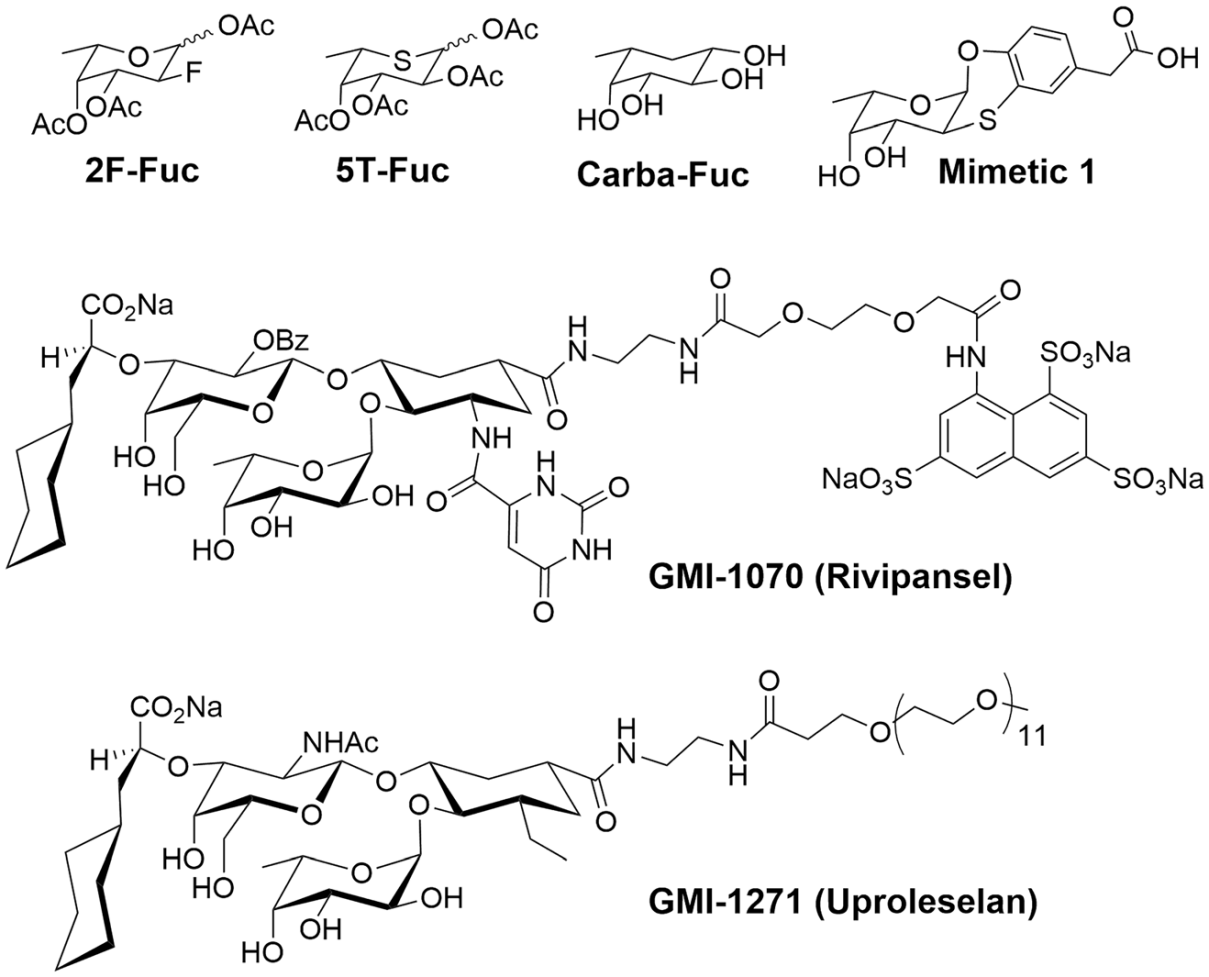
Structures of **2F‐Fuc**, **5T‐Fuc**, **Carba‐Fuc**, the fucose mimetic **1**, the pan‐selectin antagonist **GMI‐1070** (Rivipansel), and the E‐selectin antagonist **GMI‐1271** (Uproleselan).

The cell‐permeable 2‐deoxy‐2‐fluoro‐l‐fucose (**2F‐Fuc**, Fig. 4) has been reported as a potent and global metabolic inhibitor of fucosylation, and studies using this compound have measured inhibition of Le^X^ and sLe^X^ synthesis by flow cytometry [70]. **2F‐Fuc** is a pro‐drug that is readily taken up by cells. Due to the promiscuity of enzymes involved in the GDP‐Fuc salvage pathway, it is converted, inside the cell, into the corresponding donor substrate analog GDP‐2F‐Fuc. In turn, GDP‐2F‐Fuc significantly affects the expression of both terminal, sub‐terminal, and *core* fucosylations by the direct competitive inhibition of diverse FTs (*i.e*., respectively FTIV, FTVII, and FTVIII), and by shutting down the *de novo* synthesis of GDP‐Fuc (negative feedback). Thus, the **2F‐Fuc** compound is a *glycan inhibitor*, not a *glycan‐motif inhibitor*.

**2F‐Fuc** provides an example of how glycan editing promoted by a small‐molecule inhibitor of FTs can allow for the study of the biological functions of relevant fucosylated glycans, paving the way for the optimization of new glycan‐based therapeutic interventions. This compound has been widely used by glycoscientists to define the key role of fucosylated glycan motifs in a variety of biological and pathological processes in eukaryotic organisms, including cell adhesion, inflammation, tumor metastasis, immune response, sickle cell disease, and tissue development [23].

Similar results have been reported by Vocadlo and co‐workers [71, 72] with the 5‐thio‐fucose (**5T‐Fuc**, Fig. 4) and more recently the carba‐fucose (**Carba‐Fuc**, Fig. 4) derivatives. **5T‐Fuc** and the **Carba‐Fuc** are likewise metabolically converted into the nucleotide analogs GDP‐5T‐Fuc and GDP‐carba‐Fuc and broadly inhibit fucosylations. In particular, 5T‐Fuc affects the expression of fucosylated *N*‐glycans produced by FTIII and FTVII. The use of this compound results in decreased levels of cell surface sLe^X^ (sLe^X^‐specific CSLEX‐1 mAb was used) and Le^X^ in the human hepatocellular carcinoma‐derived cell line HepG2 and the human leukemia cell line HL‐60, and it impairs the adhesion of these cells to endothelial cells. In contrast to **2F‐Fuc**, no detectable changes in *core* glycosylations occur. A screening of HepG2 and HL‐60 cells in lectin‐based assays showed that the ability of **Carba‐Fuc** to inhibit α(1 → 3)‐fucosylation without any transfer of **Carba‐Fuc** on the analyzed glycans; however, the glycan‐motif specificity was not assessed. This compound was mainly proposed as an efficient chemical tool to enable the production of afucosylated antibodies (by inhibition of FTVIII).

Within this framework, the conformationally constrained fucose mimetic we call ‘mimetic **1**’ [73] (Fig. 4) has been proposed as the next generation of FT inhibitors. It represents the first example of noncompetitive and GDP‐independent inhibition of FTs, whose FT inhibition may encompass an allosteric effect. Notably, the inhibitory activity of mimetic **1** was assessed using an exofucosylation assay on both primary cells and cell lines. mAbs together with flow cytometry were used to measure the effects of inhibition of α(1 → 3)‐FTs on the expression of sLe^X^ (CSLEX‐1 mAb was used) within the glycocalyx. Mimetic **1** selectively and markedly interferes with the activity of FTVI and FTVII in the creation of sLe^x^ but has no effect on the catalytic activity of FTIX that principally mediates Le^X^ synthesis. Accordingly, mimetic **1** is capable of precision *glycan‐motif editing*.

With the context of interrupting sLe^X^‐E‐selectin adhesive interactions, in a complementary fashion, we draw attention to the competitive inhibition of this adhesive axis, pioneered by Magnani and co‐workers, that has yielded a family of small‐molecule sLe^X^ glycomimetics [74, 75, 76, 77]. To date, glycomimetics, such as **GMI‐1070** (Rivipansel) and **GMI‐1271** (Uproleselan) (Fig. 4), have been investigated [75, 76]. **GMI‐1070** is a pan‐selectin antagonist, and it binds to all three selectins (E‐, P‐ and L‐), whereas **GMI‐1271** is a selective E‐selectin antagonist. Of note, **GMI‐1271** [39, 78, 79, 80, 81] has been reported to disrupt the pathogenic leukemia cell‐endothelial ‘symbiosis’ in *in vivo* models of relapsed/refractory acute myeloid leukemia (**GMI‐1271** reached Phase III clinical trials) and it results not only in the inhibition of the formation of leukemic niches but also in the disruption of several leukemia pro‐survival pathways that are secondary to selectin‐mediated adhesion. These effects result in an increased chemosensitivity of leukemia cells. However, these small‐molecule glycomimetics are subject to efficient glomerular filtration and rapid renal clearance and, accordingly, have an extremely short biologic half‐life. Thus, these agents require frequent administration (either intravenously or subcutaneously) [82]. We view the utility of these glycomimetics in conjunction with glycan‐motif editing, since the interruption of α(1 → 3)‐fucosylation by FT inhibitors would have delayed onset but a longer biologic half‐life compared with administered glycomimetics. Accordingly, the administration of glycomimetics coincident with administration of FT inhibitors could serve to bridge the biologic onset and more durable impact of FT inhibition in decreasing sLe^X^ expression on malignant cells, especially leukemias. In so doing, the combined effects of these treatments could more completely disrupt sLe^X^‐E‐selectin adhesive interactions, further enhancing the chemosensitivity of leukemias and other malignancies.

## Concluding remarks

The future of glycan‐based therapies lies in the development of precise, context‐specific agents for modifying the biologic activity(−ies) of specific glycan motifs. These approaches can range from small‐molecule competitive inhibitors of the pertinent motif or disruption of the biosynthesis of the pertinent motif. In this regard, glycan‐motif editing is an emerging field with the potential to provide efficient therapeutic approaches for life‐threatening conditions, such as cancer. By leveraging precise control over glycan structures, scientists and clinicians can target disease mechanisms at the molecular level, offering new possibilities for personalized medicine and disease intervention. With our increasing understanding of the glycobiology of cancer, it is anticipated that accelerated innovations in the rational design of glycan‐based therapeutics strategies will ensure that, by the selective targeting of cancer‐associated glycans, will remodel cancer therapeutics and greatly improve outcomes for all patients suffering with this disease. In this framework, the regulation of α(1 → 3)‐fucosylation serves as a paradigm for ‘la dolce vita’: the life‐enhancing, beneficial therapeutic effect(s) of glycan‐motif editing.

## Conflict of interest

The authors declare no conflict of interest.

## Author contributions

BR and RS jointly wrote the manuscript: contact BR for queries regarding glycochemistry and organic synthesis; contact RS for queries regarding cancer cell glycobiology and all biomedical matters.
